# An open source Python library for environmental isotopic modelling

**DOI:** 10.1038/s41598-023-29073-2

**Published:** 2023-02-02

**Authors:** Ashkan Hassanzadeh, Sonia Valdivielso, Enric Vázquez-Suñé, Rotman Criollo, Mercè Corbella

**Affiliations:** 1 Institute of Environmental Assessment and Water Research (IDAEA/CSIC), C/ Jordi Girona 18-26, 08034 Barcelona, Spain; 2 Departament de Geologia, Universitat Autònoma de Barcelona (UAB), Edificis C, Bellaterra, 08193 Barcelona, Spain; 3 Mediterranean Institute for Advanced Studies (IMEDEA, UIB-CSIC), 07190 Esporles, Spain

**Keywords:** Hydrogeology, Hydrology

## Abstract

Isotopic composition modelling is a key aspect in many environmental studies. This work presents Isocompy, an open source Python library that estimates isotopic compositions through machine learning algorithms with user-defined variables. Isocompy includes dataset preprocessing, outlier detection, statistical analysis, feature selection, model validation and calibration and postprocessing. This tool has the flexibility to operate with discontinuous inputs in time and space. The automatic decision-making procedures are knitted in different stages of the algorithm, although it is possible to manually complete each step. The extensive output reports, figures and maps generated by Isocompy facilitate the comprehension of stable water isotope studies. The functionality of Isocompy is demonstrated with an application example involving the meteorological features and isotopic composition of precipitation in N Chile, which are compared with the results produced in previous studies. In essence, Isocompy offers an open source foundation for isotopic studies that ensures reproducible research in environmental fields.

## Introduction

Water isotopic composition is of paramount importance for decision making in many fields of study, including environmental resource management^1^. The stable water isotopes ^18^O and ^2^H are indicators of diverse aspects of the hydrological cycle. δ^18^O and δ^2^H measurements in precipitation are utilized in different meteorological and hydrological studies to identify the origin of precipitation, recognize local effects in water cycle studies, define the relative shares of water with different origins in a water body, describe aquifer recharging and characterization process and investigate various aspects of runoff and stream flow generation. All these features are essential for the optimal and sustainable management of water resources^2,3^.

The isotopic composition of rainwater is influenced by different physical variables and processes: temperature; pressure; humidity during condensation (to generate precipitation)^4,5^; mixtures of air masses with distinct origins^6^; the isotopic composition of the seawater from which air moisture condenses^7^; in-cloud microphysical processes^8–12^; the moisture conditions below clouds and the partial evaporation of precipitation along the path between clouds and the ground^13–15^; and the mixture of recycled precipitation from evapotranspiration over continents^16–18^. Therefore, detailed isotopic signature studies are used to discern these effects in any study area.

A linear relationship called the global meteoric water line (GMWL) is present between the δ^18^O and δ^2^H of meteoric water at the global scale, and this relationship is defined as δ^18^O = 8*δ^2^H + 10^14^. The characteristic isotopic signature of meteoric water in a particular region is caused by the various temperatures, relative humidity values, amounts of precipitation, latitudes and landmass proximities. The water molecules components (O, H) undergo isotope fractionation during phase transitions and the ratios of heavy versus light isotopes acts as a traceable feature of the physical processes^19–23^.

Two common approaches are available for studying the global distribution of the isotopic composition of precipitation: isotope-enabled atmospheric general circulation models (IGCMs) and regression statistics-based approaches^24^. IGCMs are numerical models that improve our understanding and reveal valuable information of the atmosphere by considering different physical processes (diffusion, advection, convection, etc.), including the physics of water isotopes (e.g., isotope fractionation, evaporation, condensation, among others)^25^. Computational power and numerical modelling advancements in recent decades have played an important role in the development of IGCMs, as they have resulted in a variety of models at different regional scales with diverse levels of complexity, such as CAM5^26–28^, ECHAM5^29,30^, MIROC^31^ and LMDZ4^32^. IGCMs are usually complex, time consuming and computationally demanding simulations. On the other hand, regression statistics-based models are generally useful in identifying the possible processes suffered by water samples based on their isotopic signature. Statistical models are simple to apply and are more intuitive to interpret. Consequently, they are used as stand-alone or complementary–preliminary tools for interpreting IGCM models and evaluating their results^25^.

Statistical models exhibit some shortcomings that can limit their usage or lower their precision. First, in contrast with IGCMs, there is no specific standalone tool that allows the user to determine the input features and databases for developing a statistical isotopic model. Second, some study areas possess scarce isotopic data or different types of isotopic samples (individual rain events versus accumulated events) and/or contain meteorological measurements with diverse spatiotemporal resolutions. This may limit the usage of the available variables that can affect statistical isotopic models^24,33^. Third, most statistical regression studies are based on simple linear models, which can neglect some of the underlying processes of the water isotopic signature by not exploring the more complex relationship between the variables. The use of both standard and novel mathematical approaches can explore these possibilities and could potentially result in discovering unforeseen aspects^34^. Fourth, the use of statistical analyses can be time- and effort-consuming, depending on the type and number of models needed or the output desired (meteoric water lines, estimation graphs, detailed maps, etc.). Automatically creating an extensive output could prevent systematic errors without compromising the possibility to carefully examine the significance and relevance of the inputs and results by the user, if it is accompanied by the informative reports of each underlying processes.

To address these shortcomings, we present Isocompy, an open source, Python-based, multistage isotopic composition analysis and modelling library. The main objectives of Isocompy are (i) to introduce an open source framework that integrates the diverse steps of stable statistical isotope modelling in a dedicated library; (ii) to incorporate novel data management, statistical analysis and machine learning regression methods accompanied by decision-making algorithms; (iii) to exhibit flexibility regarding the available input data and function with measurements that are scarce and discontinuous in time and heterogeneous in space.; (iv) to be intuitive and user friendly, which speeds up the process of forming an isotope model; and (v) to generate reports and figures in every step if needed so that the user can understand the ongoing procedure.

In the following sections, we describe the methods used (“Methods”) and the different aspects of Isocompy (“Under the hood of Isocompy”), and we demonstrate its functionality by applying it to an example involving Salar de Atacama (Chile) (“Application to the example of Salar de Atacama”).

## Methods

To create the Isocompy algorithm, bibliographical research is performed to define the innovative capabilities that would be needed for isotopic modelling. The workflow of the program is then chosen accordingly. In this section, we discuss the necessity of the capabilities that are included in Isocompy and the methodology used in the proposed workflow to form the isotopic precipitation composition models with respect to the aforementioned objectives.

Various input parameters can affect the isotopic composition of rainwater. Meteorological (precipitation, relative humidity, temperature, etc.) and geospatial parameters are two groups of input data that are widely used in isotopic modelling^35–41^. However, other information may be needed, such as sea surface temperatures, atmospheric pressures, outgoing longwave radiation (OLR) values^10,42–45^, features derived from air mass trajectories^39,46^ or features resulting from reanalysis (such as wind components, dewpoint temperatures, and evaporation values)^47^. Therefore, the workflow must allow the user to choose the nature of the input features. Moreover, in cases where the database contains unwanted data for the ongoing study, it can be modified easily.

**Figure 1:**
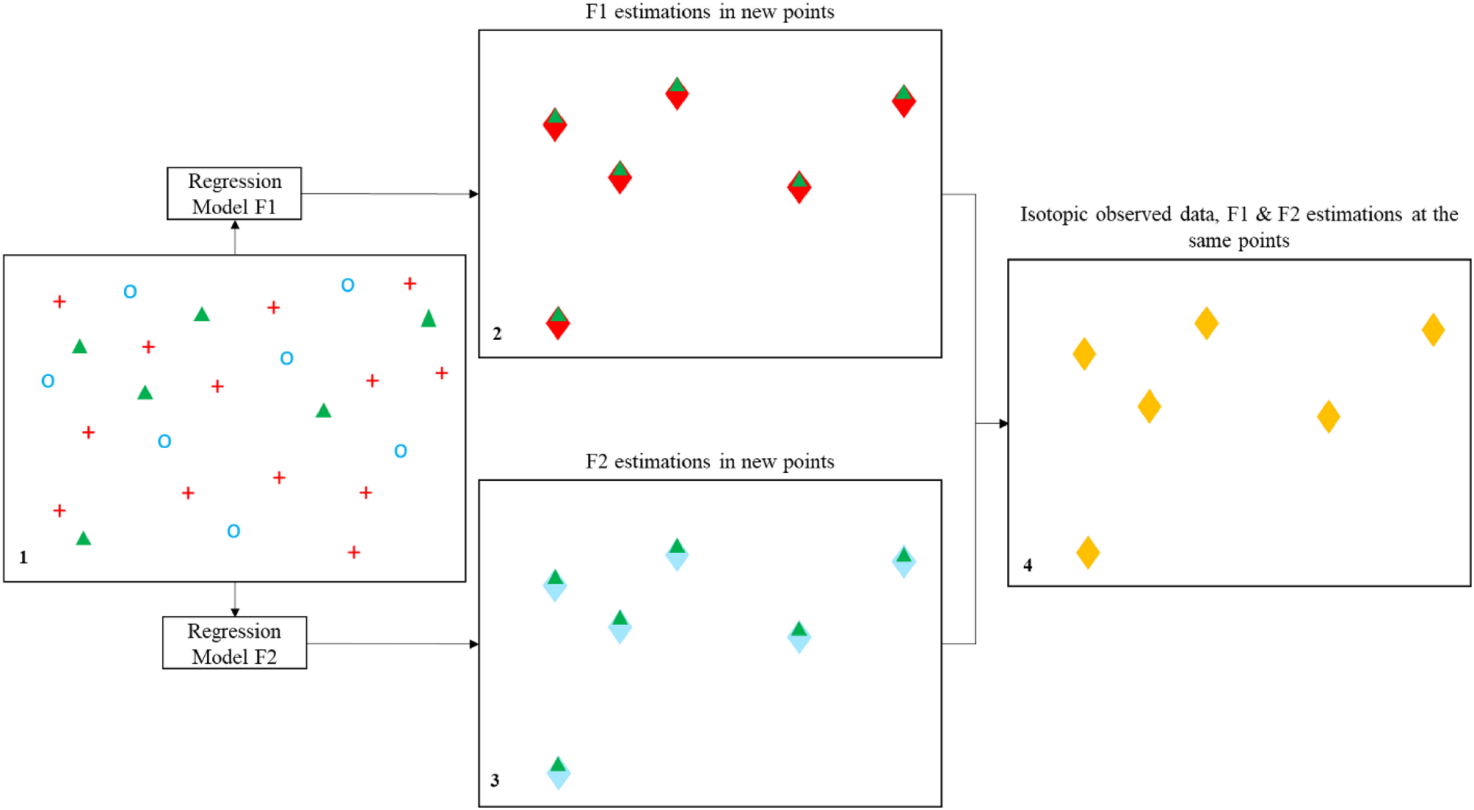
Workflow scheme for estimating isotopic values by using two independent parameters that are available in different locations than the isotopic measurements. Blue circles and red crosses represent two independent features that are potential candidates affecting the green triangles (isotopic precipitation composition). (1) Imaginary map of all available points. Green triangles are points with field isotopic measurements, and red crosses and blue circles are two independent parameter measurements. (2, 3) Estimation of the red and blue parameters from the constructed F1 and F2 regression models at the location points where isotopic data are available. (4) As a result of the algorithms, in the yellow diamonds, estimated red and blue data and measured green (isotopic composition) data are available.

Furthermore, some industrial and scientific projects are carried out in regions with limited or discontinuous spatiotemporal data. For example, in some cases, the meteorological stations are continuously maintained, which results in the production of a long-term dataset. Conversely, isotopic measurements are often sparse in time and poorly distributed in space and are not necessarily measured at the same position as other input parameters (e.g., weather parameters); this is mostly due to the complexity and costs of the analyses. Figure 1(1) illustrates an imaginary example of two independent parameters (red crosses and blue circles) that potentially affect the isotopic measurements (green triangles), but since they are not available at the same location, a one-to-one relation between the features cannot be made to perform regression. Moreover, the densities of the available data are different among the red crosses, blue circles and green triangles. To obtain of the features at the green triangle positions, first, regression models for the red and blue points, which are variables dependent on other features (in this case, geospatial features), must be generated. Figure 1(2,3) illustrate the estimations of each red and blue feature obtained at the green triangle positions derived from two separate regression models (F1 and F2, respectively).

In the yellow diamonds in Fig. 1(4), the calculated values of the red and blue parameters (estimated from F1 and F2) and the corresponding green measurements are available, which makes it possible to construct a regression model with red and blue parameters as independent variables and the green parameter as a dependent variable. By using this model, it is possible to create a map of the green parameter (isotopic composition).

Data can also vary within a time window. Utilizing the example in Fig. 1, let us assume that a continuous and dense amount of data are available for the blue parameter in a specific month during ten consecutive years. However, in the red crosses, data are available in the same month for six years. To overcome the limitations derived from different measurement frequencies and time windows, one solution is to average the 10- and 6-year measurements in the blue and red points, respectively, to obtain a single set of data for each feature in each location. Although averaging the measurements may result in a loss of information while producing less precision and higher model uncertainty, these effects would also occur with other data treatment techniques, such as filling the gaps in data series. The final workflow must also account for different parameters that are measured directly alongside the isotopic water composition or estimated via other methods, such as features derived from reanalysis^48,49^. Another important aspect of the workflow is to analyse the degrees of influence of suspected features on the dependent variable. Considering that the goal is to produce a workflow that is simple yet precise, an automatic statistical analysis procedure based on multicollinearity examination and a feature selection algorithm must be crucial parts of the workflow.

The fact that the relations between earth science variables may be linear or nonlinear suggests the capability to apply different regression methods in the workflow. The regressions must be accompanied by calibration and validation procedures to find the regression method with the highest estimation power that avoids common modelling errors such as overfitting. A total of eight regression models are considered in this study: Elasticnet^50^, Bayesian ridge regression^51^, least-angle regression^52^, Bayesian automatic relevance determination (ARD)^53^ and orthogonal matching pursuit^54^, support vector regression^55^, a random forest^56^, and a multilayer perceptron^57^. Since some of these methods are sensitive to the data scale, all inputs are standardized before applying the regressions. Hyperparameters are parameters of machine learning methods whose values control the learning process^58^. The brute-force hyperparameter search algorithm is used to obtain a suitable set of hyperparameters^59^; it is optional to fit regression methods to the transformed *ln*(*1* + *x*) of the input data alongside the original data which can potentially result in a better model in case the features have log-normal distributions. Other data transformation techniques can be applied on the input data by the user.

In Elasticnet, both L1 and L2 regularization terms are used to avoid overfitting. The Lasso (L1) and ridge (L2) regression methods are specific forms of Elasticnet regression, where the former adds the absolute value of the magnitude and the latter adds the squared magnitude as a regularization term to the cost function. Lasso and ridge regressions are achieved by introducing an L2 to L1 ratios equal to zero and one, respectively. A more detailed description of this method can be found in^50^.

The orthogonal matching pursuit method constrains the number of zero coefficients. Its residuals are calculated by using an orthogonal n-dimensional projection, which assumes, similar to independent variables, that the dependent variable can contain measurement errors^54^.

Least-angle regression is a stepwise linear regression method that moves in the direction of the most correlated feature in each step. This method is beneficial when the number of features is higher than the number of samples. Least-angle regression is sensitive to outlier data^52^.

The Bayesian ridge and Bayesian automatic relevance determination methods (also known as sparse Bayesian learning and relevance vector machine regression, respectively) form probabilistic models that include regularization parameters that are tuned according to the available data instead of being defined prior to regression^51^.

Random forest regression is a method based on the average of randomized independent decision tree estimator outputs. The main concept of this method is that the integrated final estimator may produce better results than any of the single decision trees since combining them decreases the standard deviation of the estimates^56^.

Support vector machines are versatile supervised learning methods that are used in various environmental science fields^60^. They can be used in high-dimensional environments and are flexible depending on the chosen seed functions. It must be taken into account that support vector regression can be computationally demanding^61^. Moreover, if the number of features is higher than the number of samples, the seed functions must be selected in a way that avoids overfitting^62^.

Neural networks have proven to be effective estimation techniques in various branches of science. Multilayer perceptron regression is a supervised learning method that uses L2 regularization to avoid overfitting the weights. An MLP uses a backpropagation technique. The ability to determine the number of hidden layers, the size of each layer and diverse type of activation functions mark an MLP as a flexible technique^57^. However, an MLP is complex during the process of choosing the correct estimator hyperparameters.

## Under the hood of Isocompy

### Isocompy workflow

**Figure 2:**
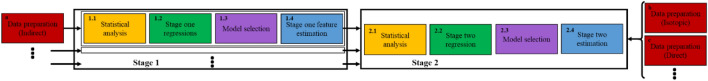
Scheme of the Isocompy workflow utilized to design the Isocompy architecture. It consists of data preparation (red boxes) and two main stages. Each stage includes statistical analysis (yellow boxes 1.1 and 2.1), regression (green boxes 1.2 and 2.2), model selection (violet boxes 1.3 and 2.3) and feature estimation steps (blue boxes 1.4 and 2.4).

Considering the abovementioned aspects of isotopic composition modelling, Fig. 2 illustrates the general scheme of our proposed workflow. It consists of data preparation and two main stages. The independent variables are introduced in the data preparation step. The goal of the first stage is to estimate the independent parameters that affect the isotopic composition model in the same space–time framework as the empirical data. The results of the first stage, accompanied by the empirical data, are incorporated into the second stage to obtain δ^18^O and δ^2^H models. Stage one of the workflow begins with a statistical analysis of the independent variables that are introduced in the data preparation step to determine their degrees of influence on the dependent variable and select the substantial variables for the regression model. The regressions are applied, and the most calibrated model is selected. Then, the variables that influence the water isotopes are estimated in the same time and space as the isotopic measurements. By preparing the data from three source groups [estimated variable data, measured variable data and measured isotopic data, (1.4, c,b in Fig. 2, respectively)], it is possible to obtain isotopic models in stage two. Again, a statistical analysis leads to the extraction of the substantial independent variables over which the regressions will be applied to select the best model. Once the models are available, the isotopic composition values can be estimated. The underlying sections of each stage are explained below.

Data preparation (the red a, b and c boxes in Fig. 2) is a key step that defines many major properties of the constructed model. Box a in Fig. 2 shows the input features named indirect features since they are not measured with isotopic values; box b represents the isotopic input measurements, and box c illustrates other features measured directly with isotopic values (direct features). In the data preparation step, different aspects of the model must be determined by the user.

- The dependent and independent variables.
- The temporal window of the input measurement choice.
- Input filtration based on specific time properties, if needed (El Niño or La Niña Southern Oscillation).
- Outlier removal based on diverse methods, if needed.
- The data averaging technique.
- Brute-force searching hyperparameter definition.

**Figure 3:**
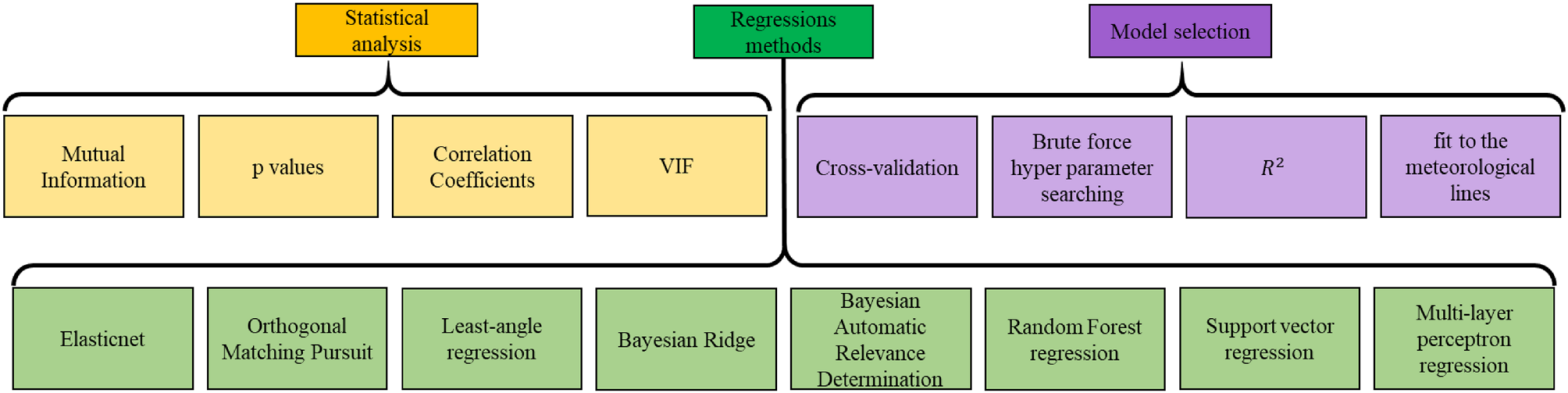
Yellow, green and violet boxes show the techniques used in the statistical analysis step, the regression methods available in Isocompy and the implemented techniques in the model selection steps, respectively.

The statistical analysis step (yellow boxes in Fig. 2) allows the algorithm to select the most considerable features to be used afterwards in the regression models. Feature selection is crucial in environmental models that normally use spatial features as inputs since autocorrelations in data may distort the estimation power of the model^63^. As shown in the yellow boxes in Fig. 3, in this stage, the algorithm calculates the p values determined by one-tailed F-test on centred data, mutual information^64^, correlation coefficients and variation inflation factors (VIFs) of the variables. The p values and mutual information help to determine the linear and nonlinear relationships between parameters and evaluate the significance of the parameters^65,66^. The VIFs and correlation coefficients are useful for detecting multicollinearity.

Since one of the main objectives of the algorithm is to facilitate and speed up the model generation process, the feature selection procedure that is derived from the statistical analysis can be performed automatically or controlled by user-defined or predefined values. In the automatic mode, the algorithm first uses the VIFs, correlation coefficients and optional pairs of features defined by the user to remove the features with multicollinearity effects that higher than a defined threshold. Then, p values are used to select the statistically significant features, based on the user-defined alpha level.

It is important to mention that since the F-test assumes that the features are distributed normally, the user have to check the normality of the features that are chosen as important features in VIF test.

The regression steps are performed in two stages of the algorithm (green boxes in Fig. 2). Various linear and nonlinear regression methods are available, as shown in the green boxes in Fig. 3, which can be selected by the user based on the nature of the given study or computational power, among other strategies. The regression methods implemented in Isocompy are described in detail in “Methods”. Nevertheless, it is worth mentioning that users with coding knowledge can add other methods of their own.

Model selection steps are also implemented in two stages of the algorithm. To find the best model, the algorithm includes and combines cross validation, brute force hyperparameter searching, R-squared fitness and goodness of fit to the GMWL or local meteoric water line (LMWL), as shown in the violet boxes in Fig. 3.

Finally, the estimation step is performed in the first and second stages, as illustrated in the blue 1.4 and 2.4 boxes of Fig. 2, by determining the substantial features determined in previous steps. This workflow ensures the flexibility of the input features, time steps and geospatial scale and, at the same time, promotes and speeds up the model generation process in an integrated algorithm.

### Isocompy architecture

The Isocompy tool examines the relationship among the input variables with various linear and nonlinear regression methods, performs a statistical analysis and dimensionality reduction, and chooses the best available regression method and its respective parameters via calibration and evaluation techniques. This is done by implementing novel machine learning, data management and statistical analysis libraries such as pandas^67^, geopandas^68^, numpy^69^, pylr2^70^, statsmodels^71^ and scikit_learn^72^. Isocompy generates extensive reports alongside figures and maps to facilitate the procedure of statistical water isotope modelling and support the user in interpreting and evaluating the results.

**Figure 4:**
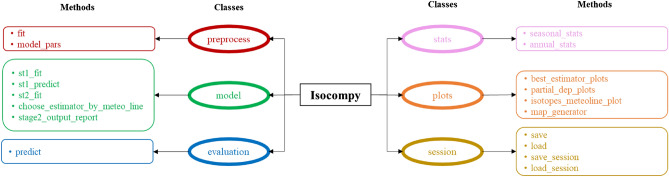
The Isocompy algorithm architecture. It contains 6 classes and 18 methods.

In this section, we describe the architecture of the underlying components and the outputs of Isocompy. It is built into six classes and 18 methods, as shown in Fig. 4. A list of the Python libraries used in Isocompy can be found in “Isocompy library information”.

### Preprocessing

The *preprocess* class holds the data preparation step (red frames in Fig. 4), whose inputs are pandas dataframes. This class has the ability to filter outliers based on upper and lower limit percentiles or modified IQR functions^73,74^. Outlier detection can be performed with or without zero values included in the data removal procedure. This is possible since there are geospatial states where zero values can result in unreasonable outlier filtration (e.g., removing the 5% lowest precipitation values from an arid zone with very few precipitation events). Data averaging can be performed based on arithmetic or geometric averaging. It is also possible to define specific time periods and limit the outputs to these episodes. Another decisive feature of *preprocess* is that the user specifies the brute-force search hyperparameters of the corresponding regression models. This selection is closely dependent on the format (i.e., volume and quality of the data) and correlation of the dataset^75^. Therefore, it is crucial that the user have an experienced-based, focused, theoretically sound, and practical search approach. Nevertheless, the default values which are described in detail in “Isocompy library information”, could be useful for dealing with complex datasets in our experience.

**Figure 5:**
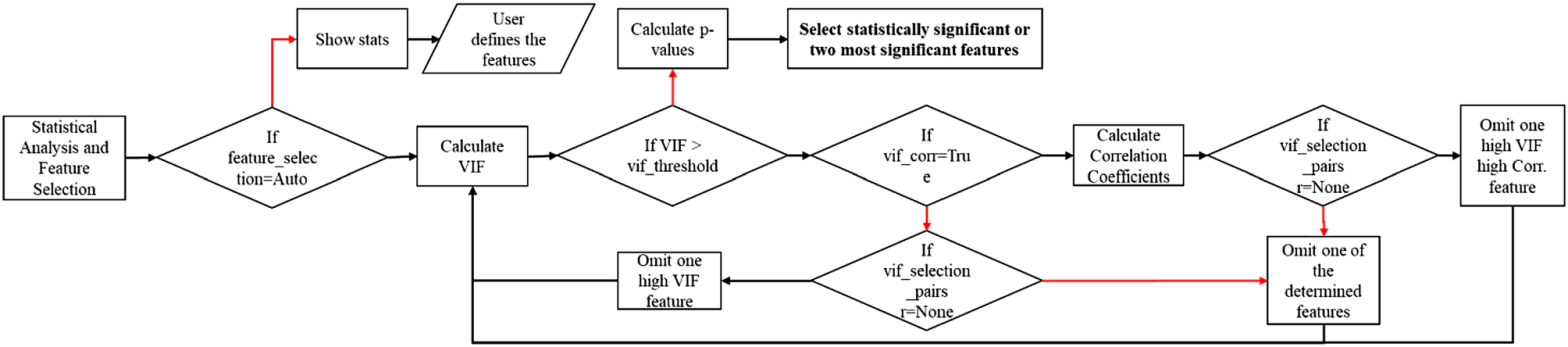
Feature selection flowchart of the model class. Red lines indicate false arguments.

The *model* class (green frames in Fig. 4) is designed to handle the statistical analysis, feature selection, model regression and model selection procedures in the first and second stages; the flowchart of this stage is shown in Fig. 5, and it can be performed manually or automatically. The statistical analysis and feature selection parameters are defined as arguments of the class. In the manual mode, the statistical analysis data are shown, and the user must choose the considerable features. In the automatic mode, Isocompy finds the parameters with the most influence on the dependent variable by comparing their correlation coefficients and VIFs with predefined thresholds in an iterative process. However, the usage of correlation coefficients, VIFs or threshold values can also be defined by the user. The output features of this statistical analysis and feature selection step (Fig. 5) feed the regression models.

**Figure 6:**
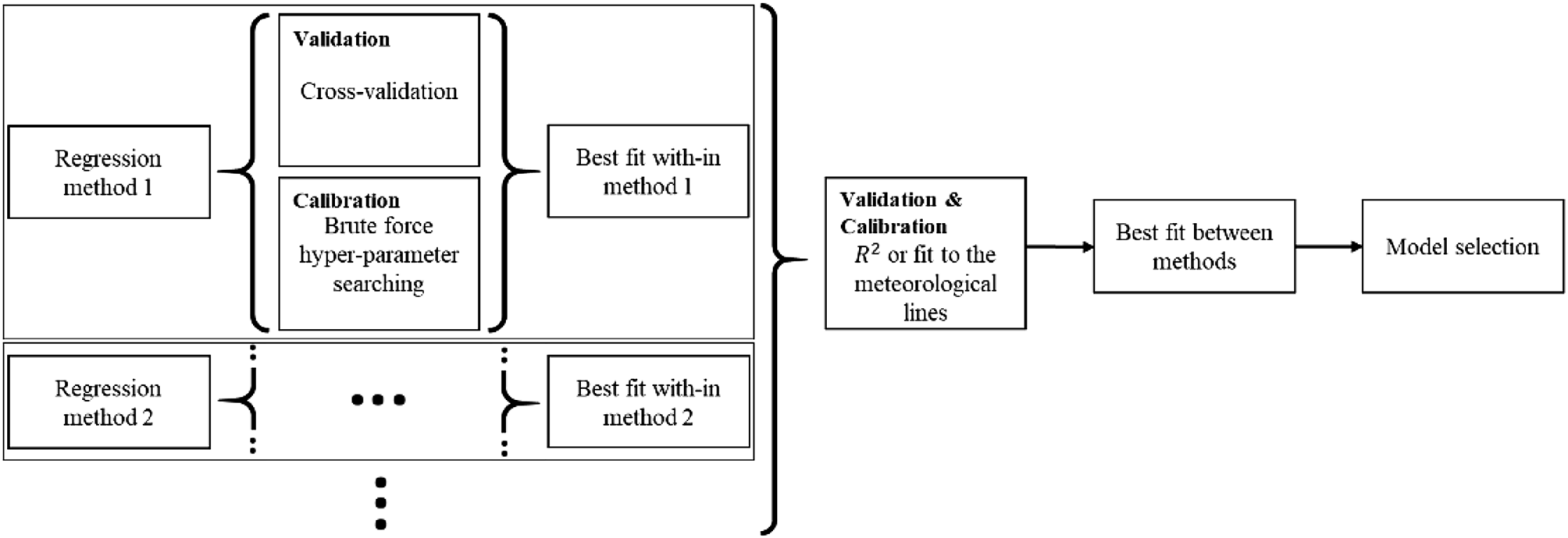
Workflow of the model regression, model validation, model calibration and best model selection processes. Black dots show that these processes are performed for each regression method selected.

Figure 6 illustrates the workflow of the regression modelling, model evaluation and calibration processes that result in selecting the best model. In each regression method, all combinations of hyperparameters are defined. For each combination, the random k-fold cross-validation technique is used to avoid overfitting. The score of a determined hyperparameter set is defined as the average score of the k models. The selected set of hyperparameters for each model is defined as the one with the highest average score. The best model among different regression methods can be selected based on preferred criteria. In the first stage, the best models are selected based on higher R-squared values, whereas in the second stage, the best model can also be selected based on three different criteria: the smallest point-to-point estimation-observation distance, the pair of models with the most similar results to the LMWL or the pair of models with the most similar results to any defined line between the water isotopes. The predefined arguments for this line are eight and ten coefficient and intercept values, respectively, that represent the GMWL. To test the different options available for selecting the best model in the second stage, it is possible to change the criteria and generate corresponding outputs.

Isocompy generates reports that include the details of all the executed models and the selected models with their R-squared values, adjusted R-squared values, VIF values, correlation coefficients, mutual information, chosen input features and sets of hyperparameters. For the chosen regression models, Isocompy also reports the cross validation averages and standard deviations obtained on the training and test data to evaluate the model estimation uncertainties.

### Model evaluation

**Figure 7:**
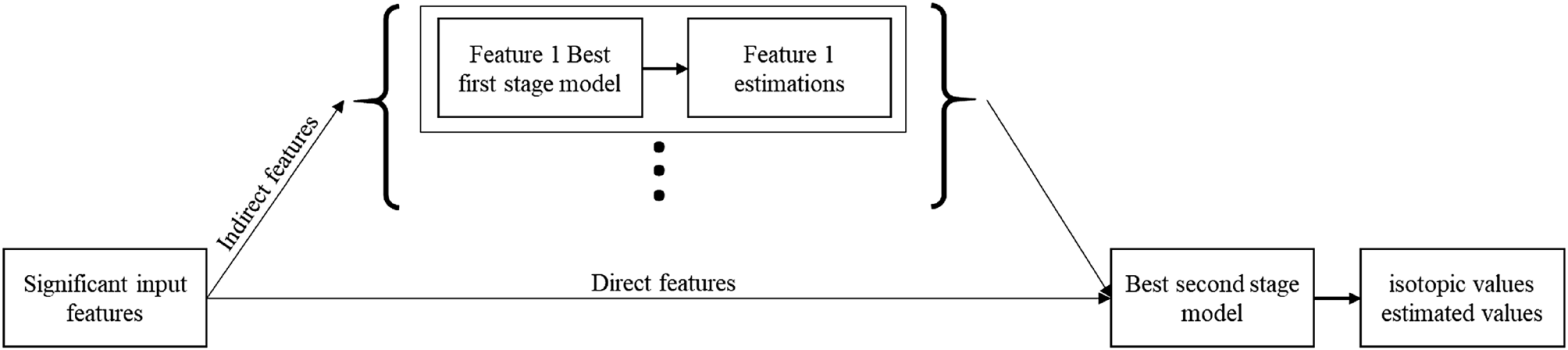
Workflow of the evaluation class for estimating the second-stage regressions.

The *evaluation* class follows the algorithm shown in Fig. 7 to calculate the outputs of the second-stage estimations. All the independent features introduced in data preparation go through the statistical analysis, and only the substantial features are used in the regression models to obtain the desired spatial–temporal estimations. Only the samples with independent determined features must be introduced, while all other data are ignored in the isotopic estimation process.

The indirect input features (box a in Fig. 2) go through the stage one estimation procedure, which are unified with the introduced direct features and are used as isotopic composition input variables for the final isotopic estimation.

### Postprocessing tools

The *stats* class generates statistical reports for each stage (pink frames in Fig. 4). They include the characteristics and details of all executed models and the selected models: their R-squared s, adjusted R-squared values, VIF values, correlation coefficients, mutual information, chosen input features and sets of hyperparameters. Isocompy also reports the chosen regression models, cross validation averages, and standard deviations of the training and test data to evaluate the model estimation uncertainties. The reports can be generated for the whole or the separate parts of the time series.

The *plot* class generates diverse kinds of graphics to illustrate the results (orange frames in Fig. 4). The shapely^76^, Bokeh^77^ and matplotlib^78^ libraries are employed to develop the methods of this class. The *partial_dep_plots* method generates partial dependency plots. The *best_estimator_plot* method constructs the plots of the best estimator in each determined time window. The *isotope_meteoline_plot* method is designed to illustrate and compare the output data and observed data with the GMWL and LMWL. This method uses the reduced major axis (RMA) regression method to calculate the local line of the input data. It is shown that the RMA approach explains water isotope relationships better than least-squares regression since it takes the measurement errors in box axes into account^79–81^. The *isotope_meteoline_plot* method can also generate residual plots of each isotopic station for each isotopic composition and accompanies them with a report including the mean absolute errors, mean square errors and means and standard deviations of the residuals, observations and estimations.

The *map_generator* method generates maps of the desired features, whether they are observed or estimated. The maps are generated based on the estimated data limits introduced by the user to the *evaluation class* in the time periods defined by the user. The results can be limited to positive values and/or to percentages if needed. The user has the ability to add a desired shapefile to the maps, display the measured data and define the aesthetics. The results can be saved as an interactive HTML file or in an image format.

### Project management

The *session* class enables the functionality of saving and loading one or all defined objects of a session (yellow frames in Fig. 4). The session class is powered by the Dill python library^82^ because of its capacity to save the executed Isocompy project as a compressed file along with its results in a single command. Hence, it would be feasible to save and close an interpreter session, send the compressed session file to another computer, open a new interpreter, decompress the session and thus continue from the point of work saved in the original interpreter session.

### Outputs

Isocompy outputs can be categorized into four groups: reports, figures, maps and datasheets. It is possible to obtain this information at different steps to clarify the underlying processes. Reports are generated to address the input data characteristics, partial and whole time period statistics, the best first- and second-stage model characteristics, all models in the first and second stages, the best second-stage model selection scoring details based on the chosen function, prediction model uncertainty statistics, residuals, observed and estimated isotopic value statistics and errors.

**Figure 8:**
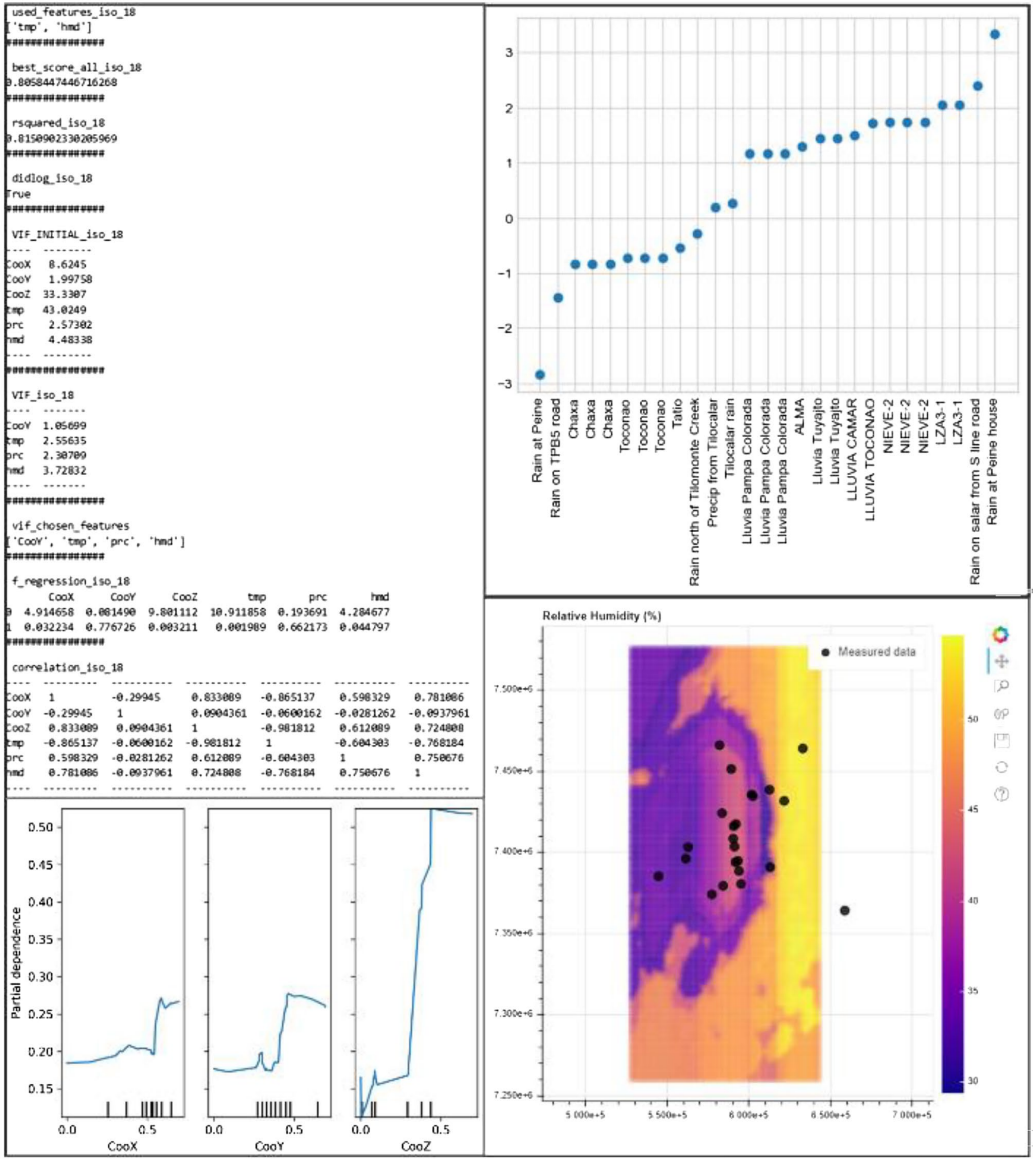
Screenshots of the outputs generated by Isocompy. Top left: an example report. Bottom left: partial dependency plots of the selected features. The values are standardized between zero and one. Vertical ticks on the x-axis illustrate the percentile of the data. Top right: a residual plot at each observation point generated by Isocompy via the *isotope_meteoline_plot* method. Bottom right: an interactive map generated by Isocompy without an available shape file.

Figures can be created for partial dependencies, observed-estimated regressions, residual plots and meteoric line plots, as explained in “Postprocessing tools”. The bottom-left and top-right parts of Fig. 8 show examples of partial dependencies and residual plots, respectively. Examples of observed-estimated plots can be seen in the figures of the next section.

Maps can be created in different formats for any desired feature by using the *map_generator* function, as mentioned in “Isocompy architecture”. Examples of maps can be seen in the figures of the next section. The bottom-right part of Fig. 8 shows a screenshot of an interactive map created by Isocompy.

Datasheets are produced in the data preprocessing stage, and they include outlier-removed data, monthly averages for each year at each station and station averages. First- and second-stage estimations are also saved in datasheets. Refer to “Application on Salar de Atacama” for an example datasheet.

## Application to the example of Salar de Atacama

The Salar de Atacama is the ideal target zone for demonstrating Isocompy capabilities due to its particular climate and topographic features. The scope of this investigation is not a comprehensive isotopic analysis, as it has been published already^83–90^, but rather validate Isocompy performance. Therefore, using the scarce information that is currently available, the climatic characteristics and isotopic composition of the precipitation in this area are compared with that of previous studies.

**Figure 9:**
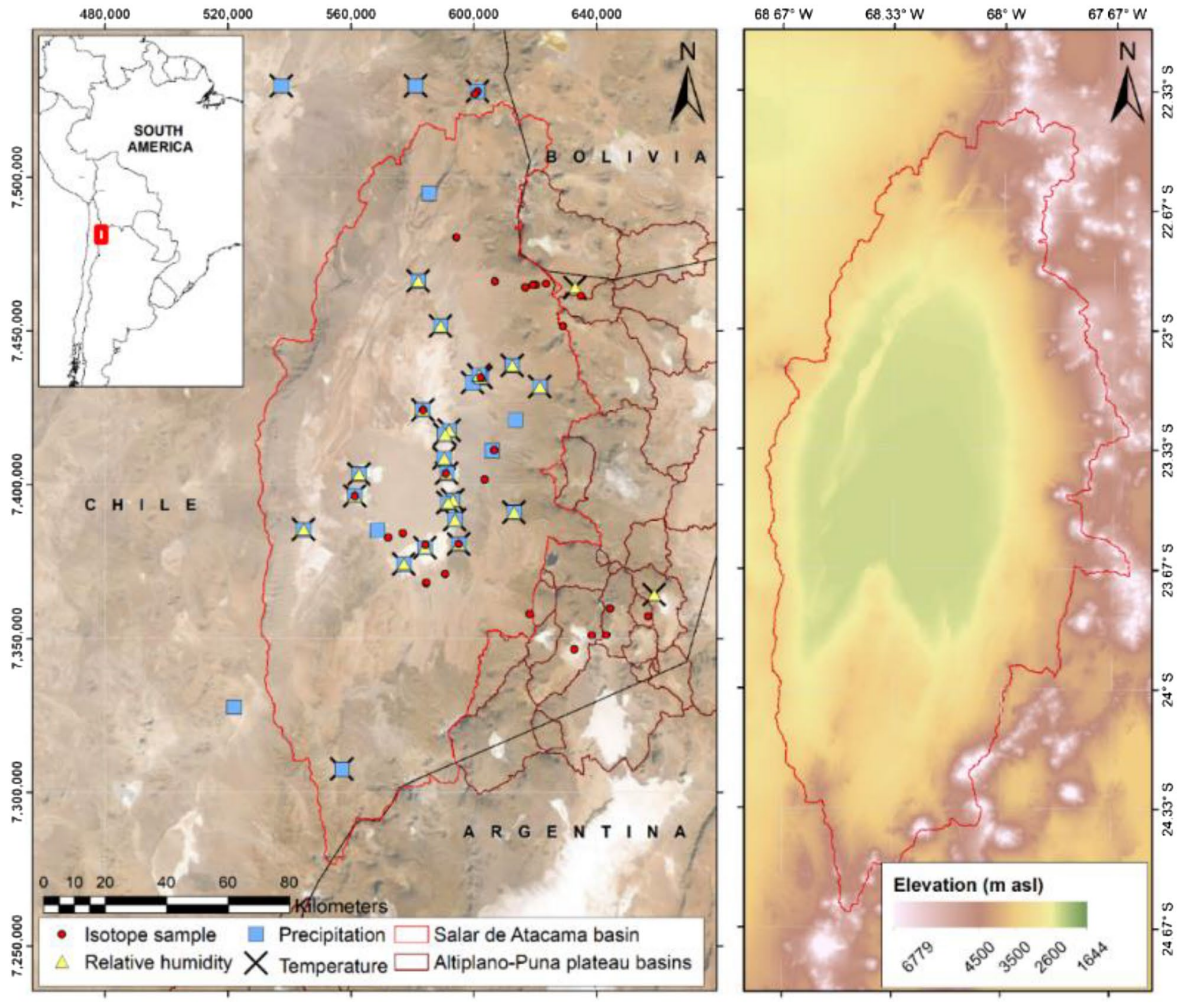
Left: location map of the study area in South America with published isotopic precipitation data (red circles) and automatic weather stations that monitor temperature (crosses), precipitation levels (blue squares) and relative humidity (yellow triangles). The solid red line delineates the Salar de Atacama basin, and the solid brown line shows the Altiplano-Puna plateau basins. The base map is derived from satellite data (SRTM from http://earthexplorer.usgs.gov/). All location data are in UTM Zone 19 S coordinates based on the WGS of 1984. The utilized DEM is an ALOS PALSAR RTC product that has a resolution of 12.5 × 12.5 m and is provided by the Alaska Satellite Facility. Right: elevation map of the Salar de Atacama basin.

The Salar de Atacama basin is located in northern Chile in the Antofagasta region (Fig. 9). This zone is the largest salt flat in Chile and the third-largest salt flat in the world. The Salar de Atacama is one of the driest places on the Earth’s surface, contains vast amounts of lithium reserves and is a valuable lagoon ecosystem (RAMSAR). For these reasons, in recent decades, many studies have been carried out on the water resources of this area^83–90^. No continuous monitoring is performed on individual precipitation events in the basin, and the available data do not have a high spatial density.

The distribution of isotopic precipitation samples is heterogeneous in time, space and type of sample. Specific rain samples are taken in the basin, and permanent rain collectors are installed close to automatic meteorological stations^91^. Isotopic samples are mostly collected during the summer months (January, February and March) since this is the period containing important precipitation events (during the so-called “Altiplanic winter”). As a result, Isocompy is applied only during these time periods.

### Input data

The available meteorological variables that potentially influence the δ^18^O and δ^2^H values in this study are air temperature, relative air humidity and amount of precipitation. They are recorded daily at the meteorological stations of the Salar de Atacama basin and its surroundings and compiled from the automatic weather stations belonging to the General Directorate of Waters^92,93^ of Chile and the Soquimich (SQM) mining company. Temperature value records are provided for the period from 1974 to 2019, relative humidity values are from 1987 to 2019 and precipitation volume data are from 1959 to 2019. The basic statistical indicators of the aforementioned meteorological variables can be seen in Table 1.

**Table 1:** Statistical indicators of temperature, relative humidity and precipitation in January, February and March for Salar de Atacama study area.

	Number of stations	Unit	Min	Max	Mean	Median	Std.dev
Temperature	28	°C	−4.3	22.6	12.8	13.2	5.5
Relative humidity	24	%	15	69.8	23.6	20.8	11.6
Precipitation	31	Mm	0	219	4.7	0	16.9

The precipitation samples for the isotopic analysis are compiled from previously published studies^94–99^ for the period from 2002 to 2021 for δ^18^O and δ^2^H and correspond to 31 different points (Fig. 9). These samples are heterogeneous: some are individual precipitation events, others are monthly accumulated or multimonth samples, and others are mixtures of rainfall and snow. The main statistical indicators of δ^18^O and δ^2^H can be seen in Table 2. Refer to “Application on Salar de Atacama” for the input data file.

**Table 2:** Statistical indicators of δ^18^O and δ^2^H in January, February and March for Salar de Atacama study area.

	Number of samples	Unit	Min	Max	Mean	Median	Std.dev
δ^18^O	52	‰ VSMOW	−15.2	−0.2	−8.1	−8.0	4.3
δ^2^H			−102.5	0.9	−53.3	−52.7	32.2

### Implementation

**Figure 10:**
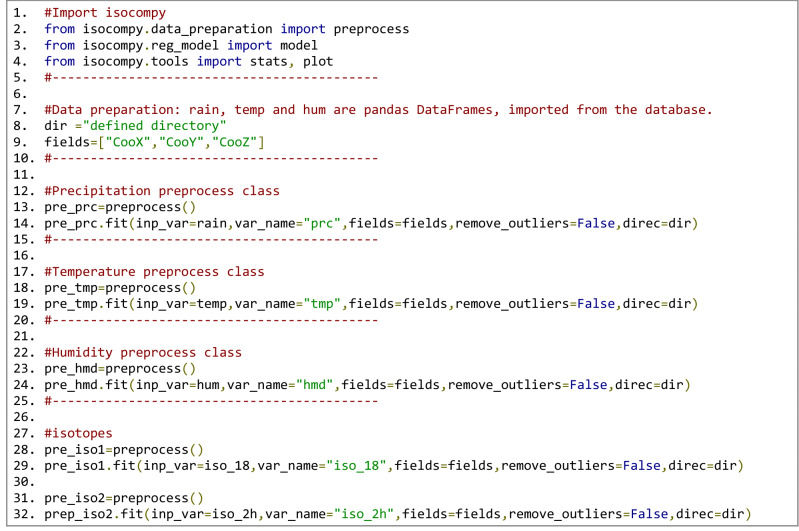
Isocompy data preparation. Location information (X, Y: coordinates; Z: altitude) is used to calculate the feature information in these positions*. Preprocess* classes are created for the precipitation, temperature, cumulative humidity, δ^18^O and δ^2^H of precipitation. *Rain*, *temp* and *hum* are panda dataframes that contain *ID*, *Date* and *Value* columns.

The data preparation steps for the input parameters are shown in Fig. 10 Lines 7 to 25 align with the *preprocess* classes for precipitation, air temperature and cumulative humidity. These three indirect variables are estimated in stage one and are dependent on the spatial variables (latitude, longitude and altitude).

Lines 26 to 32 create the *preprocess* class for the δ^18^O and δ^2^H values of precipitation used in the second stage of the model. The spatial variables here act as direct variables since they are measured in the same location as the isotopic data. In this study, outlier removal techniques, such as those explained in “Isocompy workflow”, are not needed.

**Figure 11:**
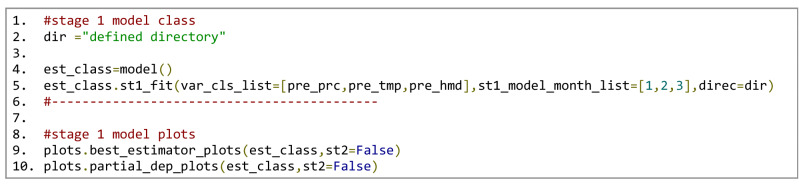
Stage-one estimation models, estimator and partial dependency plots.

The steps needed to execute the first-stage models for the desired *preprocess* classes in January, February and March of all years can be seen in Fig. 11. Each *preprocessing* class contains the dependent and independent variables, as shown in Fig. 10. The models for each process class and for each month are isolated from the rest. The feature selection options of the first stage are not changed from the predefined default Isocompy values (line 5 in Fig. 11), so the feature selection process in the first stage runs automatically. Isocompy reports the VIF and correlation coefficient values but does not consider them in the feature selection procedure. Executing lines 9 and 10 generates the estimated-versus-observed values and partial dependency plots for each regression model in stage one.

**Figure 12:**
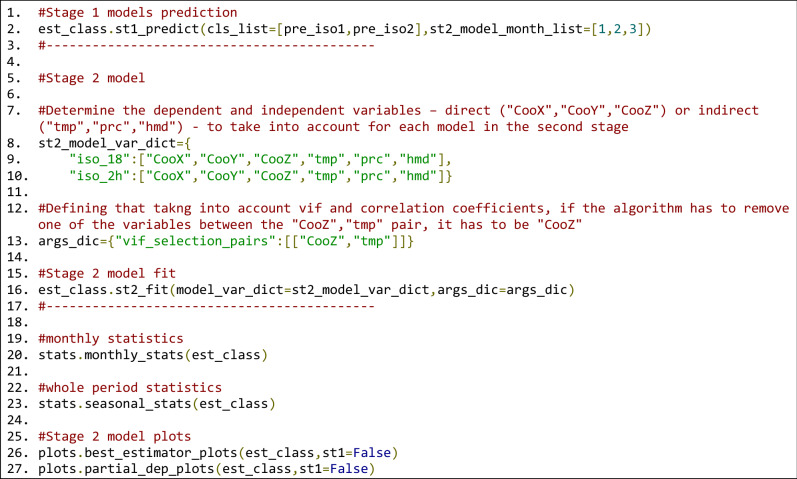
Stage-one estimation calculations (line 2). Stage-two model argument definitions (lines 7–10). Stage-two model execution (line 16). Statistical reports and plots (lines 26–27).

To create the second-stage models, the precipitation, temperature and humidity values must be predicted at the same coordinates as the isotopic measurements. Line 2 in Fig. 12 three estimates these values for three months based on the stage-one models. The dependent and independent (direct and indirect) variables must be determined as shown in lines 7 to 10.

The feature selection process of the second-stage models is the *st2_fit* method, which is performed automatically if it is not specified. In this example, some aspects of the feature selection process are specified. Thus, as seen in line 13 of Fig. 12, in cases with high VIF and correlation coefficient values, one of the parameters is removed: temperature is preferred over altitude to respect the seasonality of the data. Line 16 executes the model based on the defined variables, and lines 19 to 23 generate the statistical reports of the given month and the whole period. Similar to the first stage, lines 26 and 27 generate the estimated-versus-observed values and partial dependency plots for each generated isotopic regression model.

The reader is referred to “Application on Salar de Atacama” for the complete version of the Jupyter notebook in this study that contains the *evaluation* class, visualization options, evaluations, estimated value datasheets, meteoric lines for observed and newly defined coordinates, residual plots and feature maps.

## Results and discussion

The first stage of statistical analysis shows that altitude and longitude are significant variables for temperature and relative humidity in all 3 months, while latitude is also significant in March (Table 3). This is consistent with the DICTUC^100^ results. For precipitation, latitude and altitude are significant variables in the three summer months, as Houston and Harley^101^ mentioned, while longitude is also significant in February and March. The influence of altitude on the amount of precipitation that falls in the eastern part of the basin is recognized by all existing studies^86,102,103^.

**Table 3:** Results of the first-stage statistical analysis and models per month.

Month	Dependent feature	p-value			R^2^	Standardized standard deviation^a^	Ln (x + 1)
		Longitude	Latitude	Altitude			
January	Temperature	**7.04E−03**	6.96E**−**02	**8.06E−18**	0.98	0.23	No
	Relative humidity	**1.10E−05**	7.06E**−**02	**1.10E−05**	0.87	1	Yes
	Precipitation	2.34E**−**01	1.73E**−**01	**7.15E−11**	0.97	0.09	Yes
February	Temperature	**1.68E−03**	1.16E**−**01	**1.18E−20**	0.98	0.28	No
	Relative humidity	**8.09E−03**	9.58E**−**02	**7.01E−03**	0.84	0.13	No
	Precipitation	**6.67E−03**	**1.52E−02**	**5.74E−08**	0.96	0.68	Yes
March	Temperature	**1.80E−02**	**2.87E−02**	**2.05E−17**	0.99	0	No
	Relative humidity	**6.38E−04**	**1.14E−02**	**3.32E−04**	0.82	0.78	No
	Precipitation	2.55E**−**01	**1.03E−03**	**2.00E−06**	0.94	0.09	No

The bold p values denote significant parameters (< 0.05).

^a^Standardized standard deviation of the cross-validation scores of the estimation models.

Monthly models for temperature, relative humidity and precipitation are created by using the significant features. The estimation method with the highest scores in all models is the random forest, whose R-squared values are shown in Table 3. Column *Ln *(*x* + *1*) shows the models whose feature *Ln *(*x* + *1*) values are used since they result in higher R-squared values.

**Figure 13:**
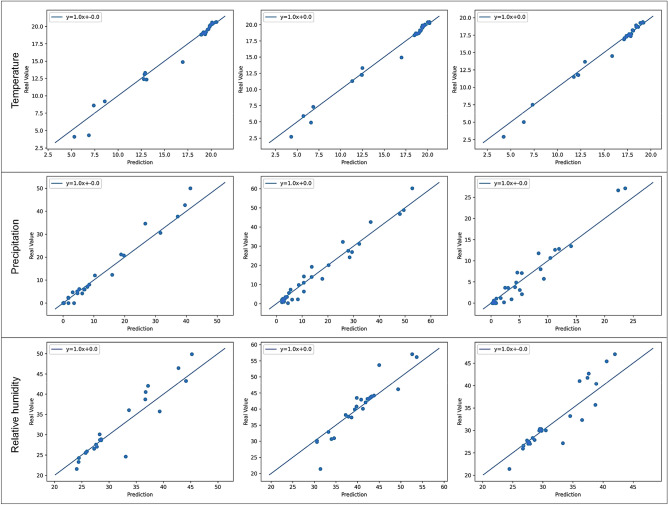
Plots of the estimated-versus-observed values generated by Isocompy for temperature, precipitation and relative humidity in January, February and March.

The estimation uncertainties can be evaluated by the standardized standard deviation of the cross-validation scores for the randomly selected test dataset in each iteration (Table 3). The limited spatial distribution of the available data in the Salar de Atacama basin can play an important role in high estimated standard deviation values obtained for some features. Figure 13 shows the observed-versus-estimated values of the three features in three months.

**Figure 14:**
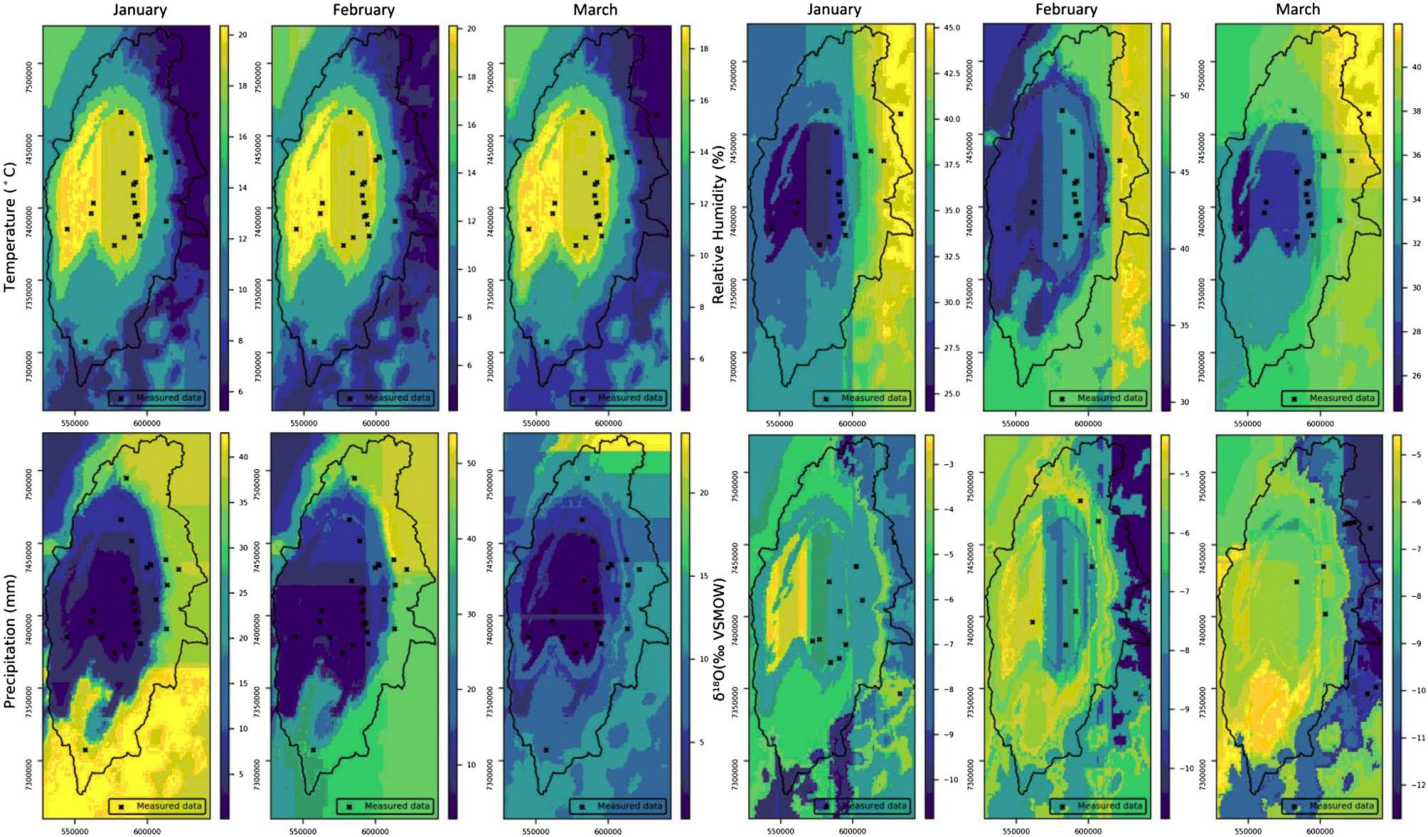
Maps of the temperature, precipitation, relative humidity and δ^18^O values of precipitation estimated by Isocompy in January, February and March in the Salar de Atacama basin.

The map of the temperature distribution estimated by Isocompy for the Salar de Atacama in the three summer months is shown in Fig. 14. It can be observed that the maximum temperatures are recorded in the central area with values between 19 and 20.4 °C, which are slightly lower than those of Marazuela et al. (24 °C) and Kampf et al. (23 °C)^102,104^ in February. It is observed that temperature decreases with altitude, reaching minimum values of 4 to 5.3 °C in the volcanic arc that surrounds the eastern side of the basin, with a gradient of approximately −0.55 °C/100 m. These gradients are similar to those presented by DICTUC and MOP-DGA (−0.56 °C/100 m and −0.65 °C/100 m, respectively)^100,105^.

The relative humidity values estimated by Isocompy in the Salar de Atacama basin for the three summer months can also be seen in Fig. 14. The lowest values of relative humidity are recorded in the core (24–29%) and in the west, and they increase with altitude, reaching their maximum values in the east of the basin (42–55%) and resulting in a gradient of 0.49%/100 m. In Valdivielso et al.^99^, who used a larger study area (N Chile), the estimated values of relative humidity in the salt flat nucleus were similar to those in the present study, although the estimated values for high altitudes were lower.

Summer storms in the Salar de Atacama basin are convective and are characterized by highly variable intensity^84,86,106,107^, with years that are much wetter than others and some with practically no precipitation. This high variability, accompanied by the nature of the available precipitation data, results in a low correlation between the precipitation values recorded in different seasons, as well as between the precipitation values recorded in the same season for different periods. Therefore, the precipitation models exhibit high sensitivity to anomalous values since they greatly affect the average precipitation at a station.

From the precipitation model, zero precipitation (0 mm) is estimated in the salt flat nucleus (Fig. 14), increasing with altitude up to 55 mm in the summits at the eastern limit of the basin; there is little precipitation at the western limits. A comparison with many studies that have presented annual isohyets maps of the Salar de Atacama^90,100,105,108^ shows that the magnitude of precipitation is lower in the present study since only the summer precipitation is considered, but the overall distribution of precipitation is similar. The summer precipitation gradient from the salt flat nucleus to the eastern peaks is 3.7 mm/100 m, which is slightly less than the annual gradients calculated in Salas et al., Valdivielso et al. (5 mm/100 m) and IDAEA-CSIC (4.6 mm/100 m)^90,91,109^, as these studies considered all precipitation events during the year. In contrast, DGA^84^ calculated values of 2.7 mm/100 m in January, 2.2 mm/100 m in February and 1.8 mm/100 m in March for the period from 1970 to 2008.

Precipitation is depleted in heavy isotopes with elevation, with an average gradient of −0.19‰/100 m in summer (Fig. 14). This gradient is slightly lower than the others calculated in this region (−0.34‰/100 m in Herrera et al. and −0.26‰/100 m in Villablanca^96,111^. The distribution map of the stable isotopic signature is consistent with the distributions of the highest temperatures, the lowest relative humidity values and precipitation in the salt flat nucleus; at higher elevations, the precipitation and relative humidity are higher, and the temperatures are lower^98,112^.

**Figure 15:**
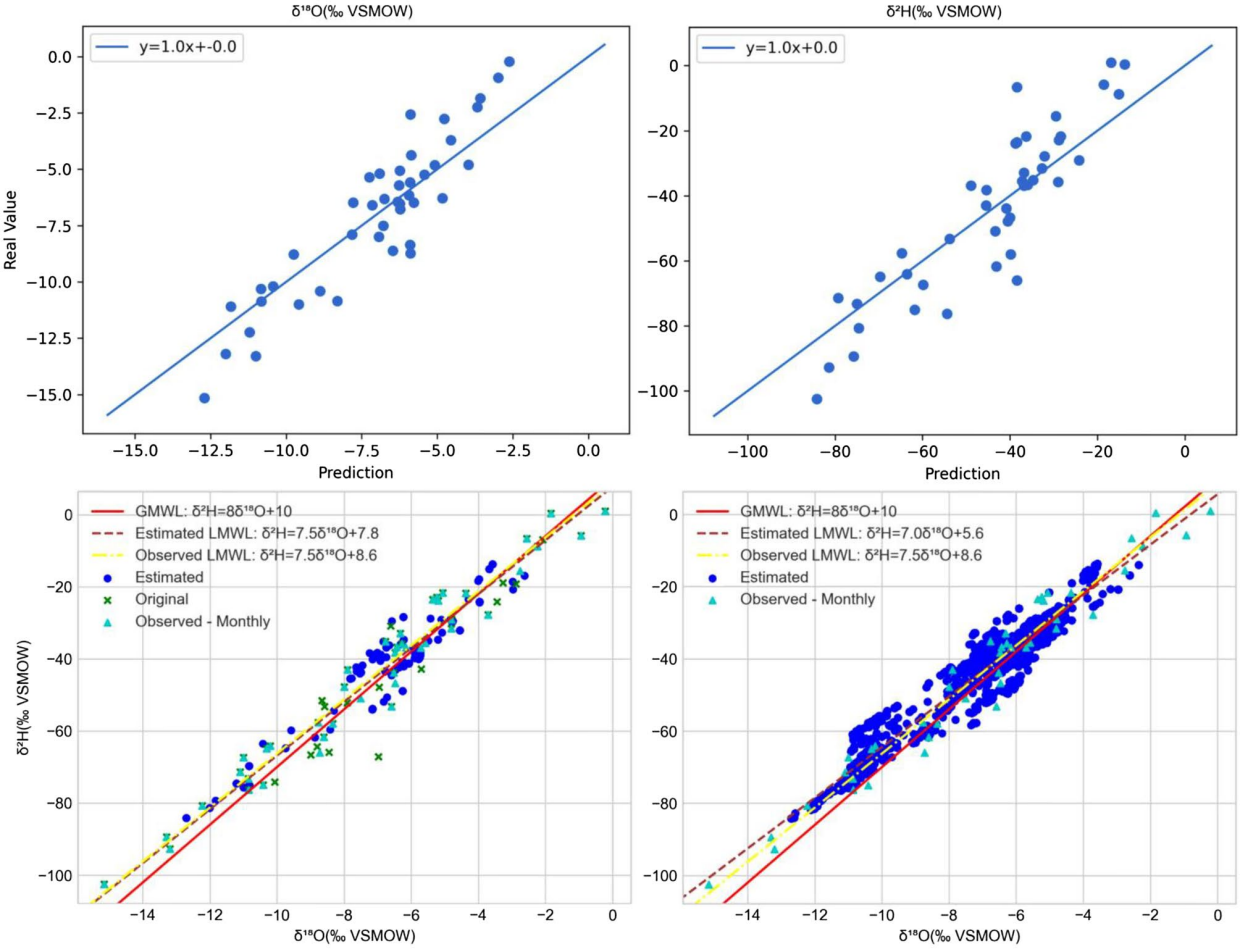
Top left and top right: estimations versus the measurements of δ^18^O and δ^2^H, respectively. Bottom: plots of the estimated (circles) and observed (triangles) δ^18^O versus δ^2^H values of precipitation. The red line is the GMWL, the brown dashed line is the estimated LMWL, and the yellow dashed line is the observed LMWL. Bottom left: the plot obtained using the δ^18^O and δ^2^H values estimated at the same points as the measurements. Bottom right: the plot obtained using the δ^18^O and δ^2^H values estimated in the study area. The reader is referred to “Application on Salar de Atacama” for the monthly meteoric line plots, residual plots and reports. All plots are generated by Isocompy.

In the statistical analysis and feature selection processes of the second stage, the initial VIF values are higher than the defined threshold (VIF = 5) for longitude, altitude and temperature. Furthermore, these variables have high correlations with each other (Table 4). Therefore, as these variables have high multicolinearity and strong correlations, altitude and longitude are iteratively removed as important features until VIF values below the threshold are reached for all the features, as seen in the VIF_fin column of Table 4. Then, the p values of latitude, temperature, precipitation and humidity are evaluated, and as a result, temperature and relative humidity are selected as significant features for the δ^18^O and δ^2^H regression models (Table 4). The R-squared values of the δ^18^O and δ^2^H estimation models are 0.82 and 0.79, and the standard deviations of the associated cross-validation scores are 0.58 and 0.46, respectively. The top-left and top-right plots in Fig. 15 show the estimation-versus-real measurements of δ^18^O and δ^2^H, respectively.

**Table 4:** The VIF values and correlation coefficients of the second-stage input features.

p-value	VIF_fin	VIF_init		Cor. lon	Cor. lat	Cor. alt	Cor. temp	Cor. prec	Cor. hum
–	–	**8.6**	Lon	1.00	–	–	–	–	–
0.78	**1.1**	2.0	Lat	−0.30	1.00	–	–	–	–
–	–	**33.3**	Alt	**0.83**	0.09	1.00	–	–	–
0.00	**2.6**	**43.0**	Temp	−**0.87**	−0.06	−**0.98**	1.00	–	–
0.66	**2.3**	2.6	Prec	0.60	−0.05	0.61	−0.60	1.00	–
0.04	**3.7**	4.5	Hum	0.78	−0.09	0.72	−0.77	0.75	1.00

VIF_init and VIF_fin show the initial and final VIF values, respectively. Cor. shows the correlation coefficients of the features. The p values of the parameters selected by the VIF process are shown.

Significant p values are displayed in bold fonts (< 0.05).

The LMWL is calculated with the isotopic measurements (observed LMWL: yellow line in Fig. 15), the average estimated δ^18^O and δ^2^H values at the same points as the measurements (estimated LMWL in Fig. 15; bottom left) and the average estimated δ^18^O and δ^2^H values in all the study areas (estimated LMWL in Fig. 15;—bottom right). Based on the estimated LMWL at the observation points, the isotopic model has slightly different slope (7.5) and intercept (7.8) values than those obtained with the LMWL defined in different areas of northern Chile^110,113–117^. However, these differences are expected since the LMWL is calculated based on a different group of points in a larger area. Figure 15 also demonstrates that the slope and intercept of the estimated and observed LMWLs are similar, which indicates that the estimated isotopic values have the same behaviour as the measured values that validates the statistical built-in capabilities of Isocompy.

## Conclusion

Isocompy is an open-source Python library dedicated to regression, statistical analysis and modelling for isotopic compositions of natural water. It considers the features that potentially affect the isotopic signature in a multistage procedure. These features can be meteorological measurements, particle trajectory-related parameters, sea surface temperatures, variables derived from reanalysis or any other parameter desired by the user.

The code simplifies and optimizes the analyses of the isotopic characteristics of natural water. The isotopic composition obtained using the Isocompy applications are consistent with those obtained in previous studies in the Salar de Atacama, which was used as an example of a study area with scarce and heterogeneous data, for validation. Therefore, Isocompy is capable of producing accurate estimated isotopic spatial distribution and estimated LMWL data. The application of Isocompy in this complex area (with unequal datasets in space and time) demonstrate the versatility on using machine learning techniques in environmental studies. Isocompy can deliver reasonable outputs, accompanied by an automatic feature selection procedure that enables a fast yet extensive study of the features that affect the isotopic composition of precipitation. The easily generated statistical analysis reports, feature maps and meteoric line plots from the observed and estimated values make the evaluation process simple and user friendly.

Nevertheless, choosing the right set of regression methods and defining a suitable set of hyperparameters for each method in a specific study area, considering the available computation power and time, is always challenging, as is selecting a suitable time window. In cases with high data densities, the number of regression models in the first stage of Isocompy can be increased by shortening the time window of each model and proceeding with the same time window in the second stage. In contrast, similar to the example of the Salar de Atacama, when the data do not have high density, it is possible to widen the time window and use data integration techniques to include more input data in the first stage, integrating the different first-stage outputs into a single model in the second stage.

Another important aspect to consider is the sensitivity of the models to anomalies in the input data. This effect is more visible when the data are scarce. Data treatment techniques such as outlier detection and data filling can be effective with Isocompy in decreasing the sensitivity of the models, but the anomalies must be considered when interpreting the results.

Although Isocompy focuses mainly on the isotopic composition of precipitation, the code could assist researchers to further environmental investigations such as paleoclimate change studies which obtaining the environmental variables from stable isotopes could be challenging. In studies where data preprocessing, statistical analysis, feature selection and machine learning are needed to investigate an environmental feature, Isocompy can be an integral solution for facilitating the workflow. In addition, Isocompy is an open-source library in a widely used programming language, which makes it a good candidate for further additions/implementations and customizations in different study areas.

Isocompy is a flexible tool that can be adapted based on the amount of data available in time and space, and it has the capability to apply diverse regression methods. It provides the user with reports, figures, datasheets and maps to facilitate the comprehension of the underlying process of each step and to speed up isotopic composition studies. Isocompy is designed to be easy to use but at the same time maintain adaptability to different studies.

### Isocompy library information

Year first available: 2022. Dependencies: pandas, pylr2, dill, geopandas, bokeh, statsmodels, numpy, tabulate, matplotlib, Shapely, scikit_learn. Contact information: ashkan.hassanzadeh@csic.es. Refer to https://github.com/IDAEA-EVS/Isocompy/wiki or https://isocompy.readthedocs.io for additional information about the installation, default values of the arguments, explanation and the usage.

### Application on Salar de Atacama

The input data, the output reports, plots, figures and maps alongside the Jupyter notebook are available free of charge in https://github.com/IDAEA-EVS/Isocompy.

## Data Availability

The datasets generated and analysed during the current study are available in the GitHub repository, https://github.com/IDAEA-EVS/Isocompy under AGPL-3.0 license.
